# Unambiguous NMR Structural Determination of (+)-Catechin—Laccase Dimeric Reaction Products as Potential Markers of Grape and Wine Oxidation

**DOI:** 10.3390/molecules26206165

**Published:** 2021-10-13

**Authors:** Stacy Deshaies, Christine le Guernevé, Lucas Suc, Laetitia Mouls, François Garcia, Cédric Saucier

**Affiliations:** SPO, Université de Montpellier, INRAE, Institut Agro, 34000 Montpellier, France; stacy.deshaies@umontpellier.fr (S.D.); christine.le-guerneve@inrae.fr (C.l.G.); lucas.suc@inrae.fr (L.S.); laetitia.mouls@supagro.fr (L.M.); francois.garcia@umontpellier.fr (F.G.)

**Keywords:** oxidation marker, (+)-catechin, phenolic NMR signals, laccase, cadmium nitrate, polyphenol oxidase

## Abstract

(+)-Catechin—laccase oxidation dimeric standards were hemi-synthesized using laccase from *Trametes versicolor* in a water-ethanol solution at pH 3.6. Eight fractions corresponding to eight potential oxidation dimeric products were detected. The fractions profiles were compared with profiles obtained with two other oxidoreductases: polyphenoloxidase extracted from grapes and laccase from *Botrytis cinerea.* The profiles were very similar, although some minor differences suggested possible dissimilarities in the reactivity of these enzymes. Five fractions were then isolated and analyzed by 1D and 2D NMR spectroscopy. The addition of traces of cadmium nitrate in the samples solubilized in acetone-*d*_6_ led to fully resolved NMR signals of phenolic protons, allowing the unambiguous structural determination of six reaction products, one of the fractions containing two enantiomers. These products can further be used as oxidation markers to investigate their presence and evolution in wine during winemaking and wine ageing.

## 1. Introduction

Polyphenols are a family of chemical compounds widely present in nature. They are found in significant amount in tea [1], cacao [2,3], blueberries [4], grapes [5], and fermented products like wine [6]. Being primary oxidation targets [7,8], polyphenols chemical structures continually evolve. These changes impact the organoleptic properties of many types of food; they are responsible for phenomena such as food browning [9] and modifications of wine’s sensory characteristics [10,11]. In enology, this oxidation phenomenon takes place in grapes or wines. Concerning enzymatic oxidation, the main enzymes responsible for browning are oxidoreductases, more precisely, polyphenol oxidase present in grapes and laccase produced by *Botrytis cinerea* [12]. 

Enzymatic oxidation mainly occurs in grape must, but further wine browning may be due to chemical oxidation reactions [7,13] or to *Botrytis cinerea* laccase that can be very stable during wine ageing [14]. Two oxidation enzymatic activities may occur on phenolic substrates: monophenol oxidase activity characterized by the hydroxylation of an existing hydroxyl group adjacent position and diphenol oxidase activity corresponding to the oxidation of *ortho*-dihydroxybenzenes to *ortho*-benzoquinones. 

According to the *Nomenclature Committee of the International Union of Biochemistry and Molecular Biology* (NC-IUBMB), these enzymatic activities are catalyzed by E.C.1-class enzymes corresponding to oxidoreductases. Among them, the three main classes of oxidoreductases catalyzing polyphenol oxidation are E.C.1.14.18.1 (monophenol monooxygenase), E.C.1.11.1 (peroxidase/POD), and E.C.1.10.3 (oxidoreductases acting on diphenols).

This last class is divided in different subclasses, and two of them appeared particularly interesting for this study: E.C.1.10.3.1 (polyphenol oxidase/PPO) and E.C.1.10.3.2 (laccase) (See Supplementary Materials Figure S1). 

PPO, laccase, and peroxidase are the oxidoreductases mainly responsible for browning during grape processing [13]. Browning caused by POD is negligible in fruits but can increase phenols degradation when combined with PPO [15]. PPO are naturally present in grapes and are able to catalyze the oxidation of monophenols to catechols and of catechols to brown pigments [8,13,16]. Laccases, occurring in *Botrytis*-infected grapes, have a wider action spectrum [17] as they can catalyze the oxidation of many different substrates. The main laccases’ oxidation targets remain 1-2 and 1-4 dihydroxybenzene.

**Figure 1:**
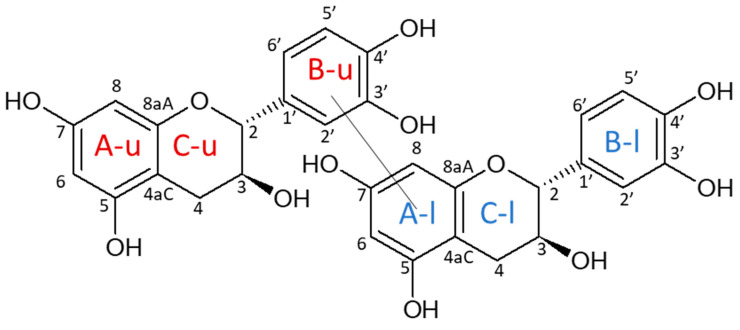
Example of a dimeric oxidation product. A, B, C rings are labelled with u for upper units and with l for lower units.

In wine, benzoquinone produced by oxidation (PPO or laccases) can easily undergo further reactions depending on their redox properties and electronic affinities [15]. They can either act as electrophiles and react with amino derivatives [18] or act as oxidants and react, among others, with phenolic substrates. Depending on their chemical conformation (quinone or semi-quinone), benzoquinone can lead to different oxidation reaction products. At a neutral pH, (+)-catechin will be oxidized to quinone on the A-ring position C5 or C7 and lead to the formation of six possible dimeric isomers implying a linkage between the B-ring position C2′, C5′, or C6′ of the upper catechin unit and the A-ring position C6 or C8 of the lower unit [19,20]. Dehydrodicatechin is a well-known product of this coupling [21]. The labeling positions of the structures are displayed in Figure 1. Under acidic conditions, semi-quinone forms can also be present on the B-ring (position OH3′ or OH4′) and lead to four possible dimeric isomers [20,22] with the upper catechin unit and the A-ring of the lower unit (position C6 or C8). Catechin enzymatic oxidation was investigated in previous studies [22,23], and the associated oxidation products were characterized by HPLC [24], though more rarely isolated and never completely characterized by NMR. 

The aim of this work was first to compare by UHPLC-MS the dimeric (+)-catechin oxidation products profiles in the presence of three oxidoreductase extracts, i.e., PPO extracted from grapes, laccase from the fungus *Botrytis cinerea* present in botrytized sweet wines [14], and laccase from *Trametes versicolor.*

The second objective was to hemisynthesize and characterize the structures of some dimeric oxidation products by NMR spectroscopy obtained with laccase from *Trametes versicolor*.

## 2. Results and Discussion

### 2.1. Comparison of Dimeric Reaction Products Profiles with Three Different Oxydoreductases and (+)-Catechin

(+)-Catechin was first oxidized in the presence of laccase from *Trametes versicolor* at pH 3.6 in the model wine solution. After separation of the dimeric fraction from residual (+)-catechin and other polymeric fractions, eight major fractions were collected and analyzed by UHPLC-UV-MS, noted from N1 to N8 in increasing retention time order (Table 1). The electrospray mass spectra in positive mode showed the ion peaks [M + H]^+^ at *m/z* 579 for N1 to N6, hypothetically corresponding to a dimer formed by a single bond between two catechin units, and [M + H]^+^ at *m/z* 577 for N7 and N8, hypothetically suggesting the formation of an additional linkage.

These eight oxidation fractions were potentially observed after the chemical depolymerization of a tannin fraction in previous works [25,26] and could possibly be the same as those already described by Guyot et al. [20], even if the experimental conditions were slightly different. Indeed, in this previous study, a crude PPO extract was used at pH 3 and 6 to obtain eight fractions. In the present study, three different enzymes were compared at pH 3.6 in the model wine solution. The LC-MS comparative analysis of the major oxidation fractions obtained with the three different enzymes (laccase from *Trametes versicolor*, laccase from *Botrytis cinerea*, and polyphenoloxidase extracted from grapes) are presented in Table 2. For each of the eight fractions, the retention times were almost identical with the different enzymes, and similar *m/z* were determined with the MS analysis. These results support the hypothesis that the same fractions were obtained for each enzyme, containing products with structures similar to those hypothesized by Guyot et al. [20]. López-Serrano and Ros Barceló [27] also performed a comparative study of the (+)-catechin oxidation products with two different enzymes: peroxidase and polyphenoloxidase, both extracted from strawberries. They concluded that the products obtained with the two enzymes were qualitatively the same. An additional compound named N4′ with *m*/*z* = 578 Th and R_t_ = 15.66 min was observed in experiments with laccase from *Botrytis cinerea* and extracted PPO but not with laccase from *Trametes versicolor*, which suggests possible differences in reactivity for these enzymes.

### 2.2. Study and Optimization of Physicochemical Parameters on ^1^H-NMR Phenolic and Aliphatic OH Signals

The structural characterization of procyanidins dimers can be obtained by NMR analysis. In particular, the precise linkage position between units may be determined using HMBC and/or ROESY correlation spectra [28,29] (Figures S2 and S3). In the case of an ether-type (C–O–C) bond, the attribution of the hydroxyl signal protons is necessary. It may also be crucial in the case of C–C linkages if some aliphatic or aromatic protons overlap or if some key correlations are missing. However, even in an aprotic solvent, the hydroxyl protons of polyphenols often appear as broad signals from which no structural information can be obtained [30]. This issue was tentatively addressed by the addition of traces of Cd(NO_3_)_2_ in the sample solutions. Indeed, ^1^H broad signals of OH groups are due to the intermolecular exchange between these OH protons and other protons in the solvent or solute. By reducing intermolecular bonds, the presence of cadmium nitrate in the samples may decrease these exchanges, thus improving the sharpness of OH proton signals.

#### 2.2.1. Effect of Cadmium Addition

**Figure 2:**
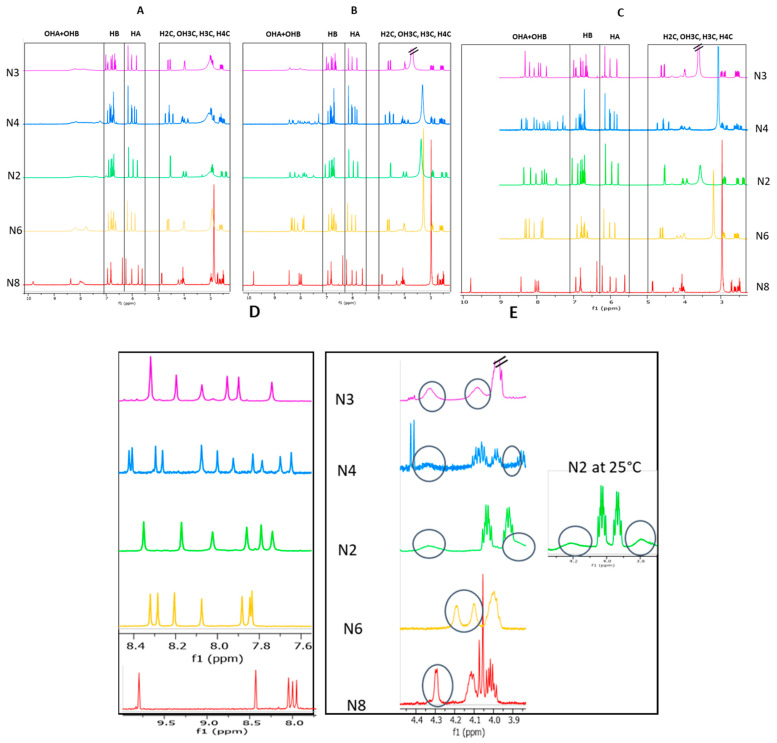
Image of 1D ^1^H spectra of the fractions N2, N3, N4, N6, and N8 at 25 °C, solubilized in acetone-*d*_6_ (**A**), at 25 °C in acetone-*d*_6_ in the presence of cadmium (**B**), at 15 °C in acetone-*d*_6_ in the presence of Cd (with an additional step consisting in dryness evaporation of the fractions N2, N3, and N4) (**C**), expansion of the phenolic (**D**) and the aliphatic (**E**) OH regions in the same physicochemical conditions as in (**C**).

After freeze-drying, the five fractions N2, N3, N4, N6, and N8 were solubilized in acetone-*d*_6_. Then, 1D proton NMR spectra were acquired at 25 °C before (Figure 2A) and after addition of small amounts of cadmium (Figure 2B). In pure acetone-*d*_6_, the phenolic OH protons of all fractions appeared as broad peaks. After the addition of cadmium, these protons showed highly resolved signals in the case of fractions N6 and N8, whereas for fractions N2, N3, and N4 the signals were only a little sharper. It should also be mentioned that increasing the Cd content had no effect upon OH signal resolution, as no sharpness or broadness of peak linewidth was observed when successive small amounts of Cd were added to the samples (data not shown).

Highly resolved phenolic OH signals from products N2, N3, and N4 were achieved thanks to additional drying and resolubilization (Figure 2C,D).

The difference of behavior upon Cd addition between the fractions may be explained by the strength of molecular interactions: stronger in the case of N2, N3, and N4 compared to N6 and N8, a further step being necessary to break these bonds.

This additional step may be the key step when using Cd to obtain highly resolved phenolic OH signals in any situation, whatever the origin of the samples, the synthesis reaction, or the natural polyphenolic products.

A previous work dealing with the unambiguous structural characterization of polyphenol dimers using highly resolved OH phenolic NMR signals thanks to cadmium nitrate addition was published in 1996 [30]. To our knowledge, no other research paper using this methodology has been published since then. Other investigations were subsequently undertaken to reach this goal, either by picric acid dosed additions [31] or by using a low acquisition temperature [32]. This may be explained by the further step necessary to get a decisive effect upon OH peak sharpness with Cd addition, as described above. However, cadmium seems to be of great value, since highly resolved signals can be obtained without the need to add precise amounts, in contrast with picric acid or NMR spectra acquisition at low temperatures.

#### 2.2.2. Effect of the Temperature

**Figure 3:**
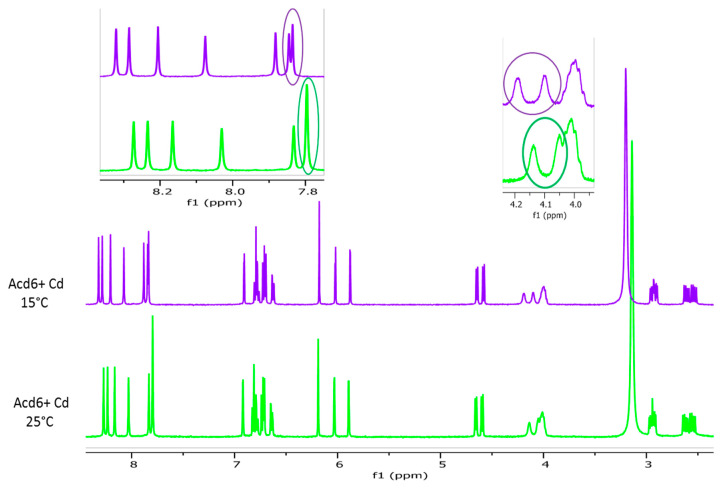
Image of 1D ^1^H spectra of fraction N6 in acetone-*d*_6_ in the presence of Cd at 25 °C and 15 °C. The expansions show the effect of temperature upon the aromatic and aliphatic OH signal chemical shifts.

A decrease of the temperature from 25 °C to 15 °C had no impact on the sharpness of phenolic OH or aliphatic OH signals. Nevertheless, downfield shifts of exchangeable proton peaks allowed us to separate some overlapping phenolic and aliphatic OH signals, making their identification more obvious (Figure 3). By decreasing the temperature, the proton exchange rate was reduced, and one might expect sharper aliphatic OH peaks [31]. The temperature of 15 °C is obviously not low enough to obtain well-resolved aliphatic OH signals. However, it allowed us to clearly identify the resonance of two aliphatic OH protons in samples N3 and N6 and of one in sample N8. The spectrum of sample N2 also exhibited two OH aliphatic protons signals, which were more distinguishable at 25 °C than at 15 °C (Figure 2E). In the case of the sample N4, the signals arising from aliphatic OH were only partly visible in the spectra, whether the temperature was set at 25 °C or to 15 °C, due to persistent overlapping (Figure 2E).

### 2.3. Structural Characterization of the Dimeric Standards—NMR Spectrum Analysis

The NMR spectra of fractions N2, N3, N4, N6, and N8 showed that the oxidation products were of high purity, since the signal intensities of other detected compounds were less than 10% compared to those of these products.

**Figure 4:**
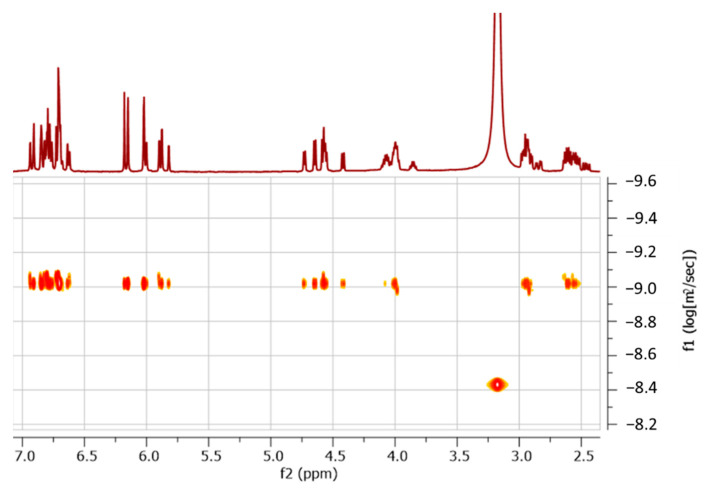
Image of 2D ^1^H DOSY spectra of a sample containing both fractions N4 and N2.

In all spectra, four ^1^H chemical shift regions typical of catechin units may be distinguish (Figure 2C): signals of the aliphatic protons of pyran rings (C rings) are found in the region from 2.3 to 5.0 ppm, and those of the aromatic signal protons of resorcinol rings (A rings) and of catechol rings (B rings) from 5.5 to 6.3 ppm and 6.3 to 7.1 ppm, respectively. The OH phenol signals of both A and B rings appeared from 7.1 to 10 ppm. The NMR spectra of both fractions showed the presence of distinct signal sets of catechin units in a constant intensity ratio: two sets of signals were observed in the spectra of fractions N2, N3, N6, and N8 in accordance with the presence of dimers, and four sets in the N4 spectra, which can correspond to one tetramer, two dimers, or a mixture of different oligomers, that is, one trimer plus one monomer. In order to determine the degree of oligomerization of the products present in fraction N4, an ^1^H DOSY experiment was performed using a mixture containing aliquots of both fractions N4 and N2. The diffusion coefficients of all signals displayed similar values (Figure 4), indicating the presence of two dimers of catechin in fraction N4.

Thanks to the fully resolved OH phenol signals which provide reliable quantitative results, the type of linkage between the catechin units may be directly deduced from peak surface area integration. Thus, for both fractions N3 and N6, the lack of one OH phenol (belonging either to a resorcinol or to a catechol ring) and the lack of one resorcinol aromatic proton indicated an interflavanic linkage (IFL) of ether type implying an O position in an A or B ring and a C6 or C8 position in an A ring. In the case of sample N2, two aromatic protons were lacking, one of a B ring and one of an A ring, implying a CA-CB IFL. The 1D ^1^H spectrum of fraction N4 showed that two protons of the B ring were lacking, as well as two protons of the A ring. The bonds between the dimer units of fraction N4 are thus both of the C–C type. The spectra of fraction N8 were quite different from the four others. Some signals were typical of catechin units, in which three OH phenols, one aromatic A ring, and one B ring protons were lacking, as well as one aliphatic OH. On the other hand, some other NMR signals are atypical of a catechin unit: a methylene with deblinded ^13^C chemical shifts (~40 ppm) and a ketone group (~192 ppm).

The proton spin systems of C, A, and B rings were determined using both ^1^H 1D and ^1^H 2D TOCSY spectra (not shown). Two ABMX C-ring spin systems (typical of catechin) were observed in the spectra of fractions N2, N3, N6, and N8, and four for fraction N4. In the spectra of fractions N2, N3, N6, and N8, two meta-coupled doublets (J~2Hz) and a singlet in the aromatic A ring region were assigned, respectively, to the A ring protons of the non-linked catechin unit and to the A ring residual proton of the of C6- or C8-linked catechin unit. In the spectra of N4, due to the presence of two dimers, four meta-coupled doublets and two singlets were detected and assigned as described above. The B ring proton systems were also easily determined from these spectra and allowed us to identify two ABM proton spin systems for the dimers of fractions N3 and N6, whereas one ABM and one AB proton spin systems were detected for dimer N2, and one ABM and one AM proton spin systems for dimer N4. The dimer N8 exhibited only one ABM B-ring spin system typical of a catechin monomer.

#### 2.3.1. Determination of the A Ring Position of the IFL of the Dimers of Fractions N2, N3, N4, and N6 

**Figure 5:**
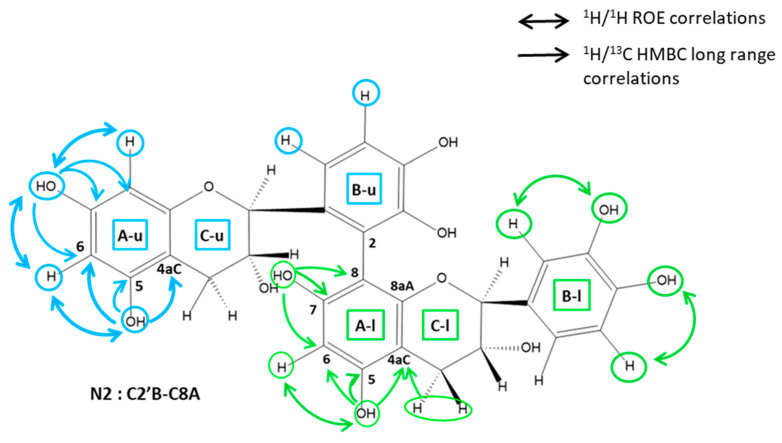

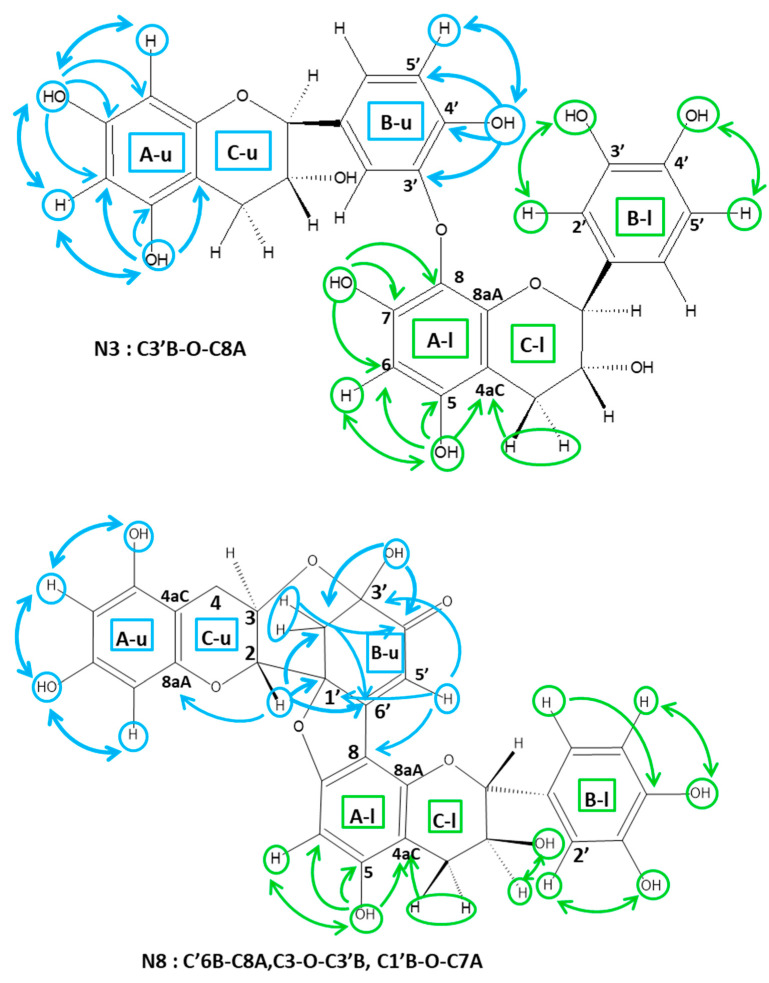
Scheme of catechin dimers (N2, N3, and N8) showing main ^1^H/^13^C long-range and ^1^H/^1^H ROE correlations, allowing linkage position determination. Blue arrows: upper unit. Green arrows: Lower units. Single arrows: HMBC correlations. Double arrows: ROEs correlations. A, B, C rings are labelled with u for upper units and with l for lower units.

The establishment of the bridge location on the A ring of dimers (i.e., C6A- or C8A-position) requires the attribution of the residual HA proton of the CA-linked catechin unit. Thanks to the highly resolved phenolic OH signals, an easy starting point was the identification of the two OH phenol protons of the A ring-linked units, i.e., the A ring that had one isolated ^1^H spin. This may be achieved using ^1^H-^13^C long-range correlations, as illustrated in Figure 5. The OH5A was readily identified thanks to a correlation with the C4aC. This quaternary carbon is indeed characterized by both its chemical shift at ~100 ppm and a long-range correlation observed with the H4C protons. OH5A also correlated with two other carbons: the most deshielded (δ > 145 ppm) was obviously C5A, while the other (δ > 125 ppm) was C6A, which also showed a correlation with the other OHA phenol proton, i.e., OH7A. This latter correlated with two other carbons: a deshielded quaternary carbon (δ > 145ppm) and a more shielded carbon (δ > 125 ppm) which were easily attributed to C7A and C8A, respectively. Once C6A and C8A are assigned, the residual HA proton may be directly attributed from the HSQC spectra. It thus was found that this residual HA proton was H6A for all fractions N2, N3, N4, N6. The IFL between catechin units thus implied a C8A position for all dimers. 

#### 2.3.2. Determination of the B Ring Position of the IFL

Dimers of fractions N2 and N4. The spectra of fraction N2 showed two different types of B ring proton spin systems: one AMX corresponding to the B ring of the non-linked unit, and one AM with a coupling constant of about 8 Hz, characteristic of H6′B and H5′B of a C2′B-linked unit. The linkage between the units of the N2 dimer is thus C2′B–C8A. The NMR spectra of fraction N4 also showed different B spin systems: two AMX, corresponding to the non-linked B-ring, and two AX spin systems, both displaying coupling constants of about 2 Hz, which are characteristic of H2′B and H6′B protons of C5′B-linked units. The presence of long-range ^1^H/^13^C correlations between H6′B and C8A, which were observed in the HMBC spectra of the two dimers, are in accordance with a C5′B–C8A linkage (Figure 5). 

Dimers of fractions N3 and N6. The spectra of fractions N3 and N6 showed the presence of two AMX B-ring proton systems and the lack of one OH phenol signal. Since all the OHA phenolic protons of the dimer units were identified (as described above), the missing OH phenolic signal can be either that of OH3′B or that of OH4′B. 

The OH position (3′B or 4′B) may be easily determined through ROE correlations with H2′B or H5′B, respectively, or using long-range HMBC correlations as illustrated in Figure 5.

The attribution of the residual OH of the B rings was readily performed using either long-range HMBC or ROESY correlations, as illustrated in Figure 5. In the case of dimer N3, a ROE correlation was observed between the H5′B and the residual OH’B of the catechin unit linked through its B ring. This OH was thus identified as OH4′B. In the case of fraction N6, the residual OH’B was assigned to OH3′B, since an ROE correlation was observed between this OH and H2′B. The long-range HMBC correlations are in accordance with these attributions. The linkage positions of these two dimers were then determined as follows: CO3′B–C8A and CO4′B–C8A for N3 and N6. respectively.

Fraction N8. Spectrum analysis of the dimer N8 showed that one unit of this dimer is a catechin with two linkage positions one the A ring, one at the C8A, and the other at the C-O7A position, since the protons H8A and OH7A are missing. The other unit of this dimer exhibited singular spectral features, indicating the loss of the B ring aromaticity and the presence of several linkage positions on both B and C rings.

The ^1^H NMR signals arising from the B ring were two doublets at 2.49 and 2.71 ppm, exhibiting a geminal coupling of ~15 Hz (12.03 ppm) typical of a methylene group and a singlet at 6.38 ppm arising from an ethylenic proton. Since these methylene and ethylene protons were not coupled, they are likely to be in positions 2′B and 5′B. The HMBC spectrum showed all correlations, allowing accurate attributions of these B ring carbons, as illustrated in Figure 5. The H2C of this unit gave three correlations with B ring carbons: one is the methylene carbon at ~45 ppm, which was thus attributed to C2′B, and the remaining two, with carbons resonating at ~90 ppm and ~162 ppm, which can be assigned to C1′B and C6′B. H5′B gave only strong ^3^J correlations with two quaternary carbons of this B ring: one is the carbon previously assigned to C3′B (~95 ppm), and the other one, which resonated at ~90 ppm, could thus be attributed to C1′B. The carbon at ~162 ppm was then deduced to be C6′B. 

The presence of an aliphatic OH (~5.8 ppm) at the C3′B position (~95 ppm) was determined through its ROE correlation with both H2′B protons. Furthermore, OH3′B gave HMBC correlation with a quaternary carbon at ~192.5 ppm, characteristic of a ketone group at the C4′B position.

The shielding of this C1′B of about 40 ppm is in accordance with a loss of the B ring aromaticity. Furthermore, the lack of OH at the C7A position of the other unit is in agreement with an ether linkage C1′B–O–C7A.

The NMR data showed that the C ring of this unit does not have any OH3C. The presence of a C3C–O–C3′B linkage is in accordance with the shielding of C3C of about 1.5 ppm as well as the chemical shift of C3′B which is typical of a hemiketal carbon (95 ppm).

Altogether, the NMR spectral data allow us to conclude that this dimer corresponds to the dehydrocatechin A described earlier by Weinges et al. [33] and then by Guyot et al. [20].

**Figure 6:**
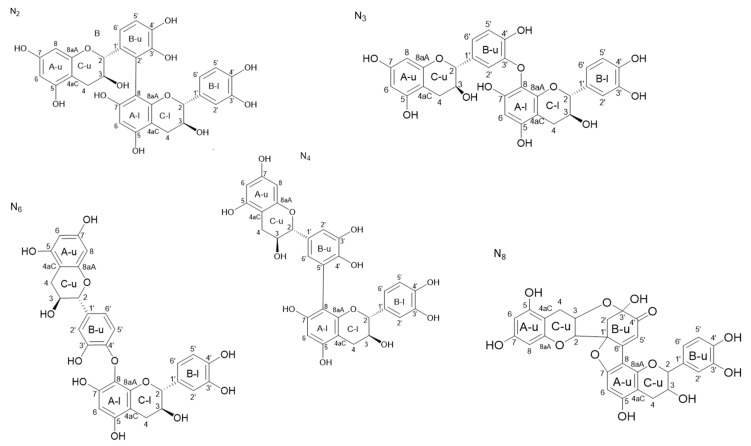
Structures of the six dimeric oxidation products formally identified by NMR analysis, N4 corresponding to a mixture of two isomers. Upper units rings are labelled with u and lower units rings are labelled with l.

The structures of the six dimeric compounds determined by these NMR analyses are shown in Figure 6, N2, N3, N6, and N8 being pure products, and N4 being a mixture of two isomers.

## 3. Materials and Methods

### 3.1. Chemicals

(+)-Catechin hydrate ≥ 98%; laccase from *Trametes versicolor* (0.94 U·mg^−1^); sodium phosphate dibasic dihydrate ≥ 98%; citric acid (ACS reagent, cadmium nitrate tetrahydrate 99.997%; formic acid and Amberlite XAD7HP were obtained from Sigma-Aldrich) (Saint-Louis, MO, USA). Acetone-*d*_6_ was purchased from Euriso-top (Saarbrücken, Germany), and trifluoroacetic acid (TFA) from Roth Labo (Karlsruhe, Germany). Water LC-MS, acetonitrile LC-MS (ACN), and methanol LC-MS (MeOH) were all from VWR (Radnor, PA, USA).

### 3.2. Preparation of the Model Wine Solution

The model wine solution was an ethanol/water solution (12/88; *v*/*v*) with 0.033 M tartaric acid, adjusted to pH 3.6 with NaOH 1 M [34].

### 3.3. Crude Grape PPO Extracts

The PPO extract was prepared as described previously by Singleton et al. [35]. Frozen grapes were first mixed in an acetate buffer (1.5 M, pH 5; 10 g·L^−1^ ascorbic acid). The mixture was then filtered and centrifuged (3000 g; 10 min). The residue was finally washed with acetone (80%) and air-dried.

### 3.4. Laccase from Botrytis Cinerea

Laccase from *Botrytis cinerea* was obtained as described by Quijada-Morin et al. [36]. It was produced from the VA612 strain (collected in 2005 in a vineyard in Hautvillers, Champagne, France, from the Pinot Noir cultivar). Briefly, cultures on solid malt yeast medium were left for one week at 24 °C under blue light. The spores were then scraped and inoculated into a 500 mL Erlenmeyer flask containing 125 mL of culture medium (40 g·L^−1^ glucose, 7 g·L^−1^ glycerol, 0.5 g·L^−1^ l-histidine, 0.1 g·L^−1^ CuSO_4_, 1.8 g·L^−1^ NaNO_3_, 0.5 g·L^−1^ KCl, 0.5 g·L^−1^ CaCl_2_·H_2_O, 0.05 g·L^−1^ FeSO_4_·7H_2_O, 1.0 g·L^−1^ KH_2_PO_4_, and 0.7 g·L^−1^ MgSO_4_·7H_2_O). After 3 days of incubation and 2 days of growth in the same previous medium, gallic acid (2 g·L^−1^) was added to the pre-cultures. After 5 days, the liquid medium was filtered, and the supernatant was submitted to tangential filtration in a Quixstand filtration system (GE Healthcare UK, Little Chalfont, England) equipped with a 30 kDa-molecular weight cut off membrane. The concentrate was finally subjected to a diafiltration against distilled water, and only the fractions that presented oxidant activity against ABTS were kept (−80 °C).

### 3.5. Oxidation Procedure

A laccase solution (1 g·L^−1^) in phosphate-citrate buffer was previously prepared and added to a 6 g·L^−1^ (+)-catechin solution (model wine) to obtain a laccase final concentration of 0.3 g·L^−1^. The obtained solution was then slowly stirred (180 rpm) at room temperature for 2 h. The concentrations were previously optimized, and the experimentation was performed in triplicate.

### 3.6. Reaction Stopping on Resin Amberlite XAD7HP

An amberlite column was conditioned with ethanol (absolute) and rinsed with two column volumes of milli-Q water. The previous laccase/(+)-catechin reaction medium was dropped on the column and first eluted with two column volumes of milli-Q water [37]. The column was then eluted with ethanol until the collected fraction was uncolored. Only ethanol fractions were kept, evaporated, and lyophilized. The powder was stored at −80 °C until used.

### 3.7. Purification Procedure of the Dimeric Fraction Using Flash Chromatography

The lyophilized powder was first purified using a flash chromatography system puriflash430 equipped with a UV detector set at 280 nm and a Puriflash diol 50 µm f0025 column. The binary mobile phase consisted of acetonitrile (solvent A) and methanol (solvent B), both acidified with 0.1% TFA. A series of injection were performed at a constant flow rate of 20 mL·min^−1^, using the following gradient: 100% A for 4.4 min; 0–10% B in 10 min; 10% B for 5 min; 10–90% B in 5 min; 90% B for 3 min; 90–10% B in 2 min; 10% B for 10 min. The injection volume was 1 mL (300 mg lyophilized powder dissolved in 1 mL of solvent A). Three distinct fractions were collected each time. The first one corresponded to residual (+)-catechin, and the third one was a mixture of high-molecular-weight polyphenols. The second eluted fraction, containing a mixture of dimeric oxidation products, was evaporated and lyophilized before the second purification step.

### 3.8. Purification Procedure of Oxidation Products from the Dimeric Fraction Using a Semi-Preparative Chromatographic System

The fraction containing dimeric oxidation products was purified using a semi-preparative Bio-Rad NGC 10 medium-pressure chromatography system equipped with a reversed-phase Varian Dynamax C18 Microsorb column (250 × 21.2 mm; 3 µm). The binary mobile phase consisted of milli-Q water (solvent A) and 80% acetonitrile, 20% Milli-Q water (solvent B), both acidified with 0.05% TFA. A series of injections (300 µL) of the lyophilized powder (20 mg dissolved in 200 µL of solvent A and 100 µL of ACN) were performed under the following elution conditions: 100% A for 4 min; 0–35% B in 46 min; 35–100% B in 2 min; 100% B for 5 min. Eight distinct fractions were collected each time, corresponding to pure UPLC signals at 280 nm. Each fraction was evaporated and lyophilized before NMR analysis.

### 3.9. Sample Preparation for NMR Analysis

About 1 mg of each lyophilized powder weighted in Eppendorf tubes was solved into 500 µL of acetone-*d*_6_. Then, ~10 µL of a concentrated solution of cadmium nitrate in acetone*-d*_6_ was added to the samples, and the resulting solutions were transferred to 5 mm NMR tubes for NMR analysis. An additional step was performed for some samples: after solubilization of the lyophilized powders in acetone-*d*_6_ in the presence of traces of cadmium, the samples were evaporated to dryness and then re-solubilized in acetone-*d*_6_ without further addition of Cd.

### 3.10. Instrument Specifications

UPLC-MS analysis. The reactions were monitored using two UPLC-MS systems. The first one was used to precisely identify products’ retention times using a long gradient method. i.e., Waters reversed-phase Ultra-High-Performance Liquid Chromatography coupled to Mass Spectrometry (UHPLC-MS). The liquid chromatography system was an Acquity UPLC (Waters, Milford, MA, USA) equipped with a photodiode array detector. We used an Acquity UPLC HSS T3 column (1.8 µm, 2.1 × 150 mm). The column temperature was 25 °C. The binary mobile phase consisted of 0.1% formic acid in water (solvent A) and acetonitrile (solvent B). The separation was performed at a constant flow rate of 0.25 mL·min^−1^, using the following gradient: 8–11% B in 2 min; 11% B for 8 min; 11–25% B in 15 min; 25–55% B in 5 min; 55–99% B in 1 min; 99% B for 4 min; 99–8% B in 1 min; 8% B for 4 min. The injection volume was 5 µL. The mass spectrometer was a Waters Acquity QDa electrospray ionization (ESI) simple quadrupole (Waters, Milford, MA, USA). The capillary voltage was set at 0.8 kV. The mass spectra were acquired over a mass range of 200−900 Th in the positive ion mode.

The second UHPLC-MS system, used for a rapid verification during the purification steps, was the same as described previously, with an Acquity UHPLC HSS T3 column (1.8 µm, 2.1 × 100 mm) heated at 38 °C. The separation was performed at a constant flow rate of 0.55 mL·min^−1^, using the following fast gradient: 0.1–40% B in 5 min; 40–99% B in 2 min; 99% B for 1 min; 99–0.1% B in 1 min. The injection volume was 2 µL. The mass spectrometer was a Bruker amaZon X electrospray ionization (ESI) ion trap (Bruker Daltonics, Bremen, Germany). The capillary voltage was set at −5.5 kV. The mass spectra were acquired over a mass range of 50−2000 Th in the positive ion mode. 

All UPLC-MS analyses were performed in triplicate.

NMR Instrumentation. All the NMR spectra were recorded on an Agilent DD2 500 MHz spectrometer (Agilent Technologies, Santa Clara, CA, USA), operating at 500.05 and 125.74 MHz for proton and carbon-13 nuclei, respectively, using a 5 mm indirect detection probe equipped with a z gradient coil. 1D ^1^H and ^13^C, 2D homonuclear ^1^H TOCSY and ROESY, and heteronuclear ^1^H/^13^C HSQC and HMBC experiments were performed using classical pulse sequences and analyzed using both VNMRJ4.2 and MestReNova 14.2.1 (Mestrelab Research, Spain) software. DOSY measurements were acquired and processed as previously described [38]. The acquisition parameters of the DgcsteSL pulse sequence were as follows: the diffusion delay time and the gradient pulse width were set at 50 ms and 2 ms, respectively, the gradient strength (g) was incremented in 16 steps with equal g2 spacing from 0.3 to 32 G·cm^−1^. After phase correction, 2D DOSY spectra were constructed from the peak height measurement using VNMRJ4.2 software. 

All spectra were referenced to the solvent acetone-*d*_6_ signals (^1^H residual signal at 2.05 ppm and ^13^C signal at 29.92 ppm).

## 4. Conclusions

The action of three different oxidoreductases (polyphenoloxidase extracted from grapes, laccase from *Botrytis cinerea*, and laccase from *Trametes versicolor*) on (+)-catechin were investigated, and the LC-UV-MS resulting profiles were very similar, although some minor differences suggested possible dissimilarities in the reactivity of these enzymes.

The structures of six catechin-laccase oxidation products (using laccase from *Trametes versicolor*) were obtained on the basis of specific NMR signatures (four pure products, i.e., N2, N3, N6, and N8, and N4, corresponding to a mixture of two isomers). The complete attribution of phenolic OH signals was possible thanks to the addition of cadmium nitrate with a simple preparation procedure that allowed the unambiguous attribution of the linkages between the catechin units for some of the compounds of interest. This procedure will greatly simplify NMR analysis of polyphenols mixtures, either synthetized or extracted from natural products. 

The standards obtained in this work may be used in the future as oxidation markers to investigate their presence and evolution during grape ripening and wine ageing. Besides catechin, other polyphenol compounds, including flavonoids and non-flavonoids, may also be used as substrates of laccase to obtain additional new standards.

## Figures and Tables

**Table 1:** Analytical reversed-phase UHPLC retention times, absorbance maxima, corresponding *m/z* (Th), and yields (%) for the eight major oxidation products collected.

Compound	R_t_ (min)	λ_max_ (nm)	*m*/*z* (Th)	Molar Yield (%)
N1	6.14 ± 0.02	280	579	0.4
N2	6.91 ± 0.02	280	579	0.1
N3	10.34 ± 0.03	280	579	0.7
N4	14.11 ± 0.07	280	579	0.3
N5	17.13 ± 0.02	280	579	0.2
N6	19.03 ± 0.01	280	579	0.7
N7	20.63 ± 0.03	263, 318	577	0.1
N8	25.23 ± 0.01	256, 280, 286	577	0.2

**Table 2:** Qualitative comparison of analytical reversed-phase UHPLC retention times for the eight major oxidation products with the three different oxidative enzymes: laccase from *Trametes versicolor*, laccase from *Botrytis cinerea*, and polyphenoloxidase extracted from grapes. The results are expressed as mean values (*n* = 3) with standard deviation.

	R_t_ (min)		
Compound	Laccase from *Trametes versicolor*	Laccase from *Botrytis cinerea*	Polyphenoloxidase Extracted from Grapes
N1	6.14 ± 0.02	6.14 ± 0.02	6.12 ± 0.01
N2	6.91 ± 0.02	6.86 ± 0.01	6.80 ± 0.01
N3	10.34 ± 0.03	10.29 ± 0.02	10.29 ± 0.01
N4	14.11 ± 0.07	14.10 ± 0.01	14.04 ± 0.05
N4′	/	15.67 ± 0.01	15.66 ± 0.05
N5	17.13 ± 0.02	17.12 ± 0.04	17.11 ± 0.06
N6	19.03 ± 0.01	19.00 ± 0.003	19.00 ± 0.03
N7	20.63 ± 0.03	20.59 ± 0.01	20.60 ± 0.03
N8	25.23 ± 0.01	25.21 ± 0.02	25.22 ± 0.03

## Data Availability

The data presented in this study are available in the present article and Supplementary Information Section.

## Supplementary Materials

The following are available online, Figure S1: Classification of enzymes responsible for enzymatic browning; Table S1: ^1^H and ^13^C NMR assignments for compounds N2, N3, N4, N6, and N8; Figure S2: 2D ^1^H/^13^C HMBC, top 1D ^1^H, side 1D ^13^C spectrum (a) N2; (b) N3; (c) N4; (d) N6, and (e) N8; Figure S3: ^1^H 2D ROESY spectrum showing correlations (in blue) between phenolic and aromatic protons, ROE correlations between phenolic protons due to chemical exchange appear in red (a) N2, (b) N3, (c) N4, (d) N6, and (e) N8.

## Sample Availability

Samples of the compounds are not available from the authors.

## Abbreviations

NMR: nuclear magnetic resonance, Cd: cadmium, TOCSY: total correlation spectroscopy, ROESY: Rotating-frame nuclear Overhauser effect spectroscopy, HSQC: heteronuclear single-quantum correlation experiment, HMBC: heteronuclear multi-bond connectivity, DOSY: diffusion ordered spectroscopy.
