# Supplementary material for: Unambiguous NMR Structural Determination of (+)-Catechin—Laccase Dimeric Reaction Products as Potential Markers of Grape and Wine Oxidation
Source: Molecules. 2021 Oct 13;26(20):6165. doi: 10.3390/molecules26206165 (PMC8537594; doi:10.3390/molecules26206165)

Supplementary Materials for

Unambiguous NMR Structural Determination of (+)-Catechin-Laccase Dimeric Reaction Products as Potential Markers of Grape and Wine Oxidation.

Stacy Deshaies, Christine Le Guernevé, Lucas Suc, Laetitia Mouls, François Garcia

and Cédric Saucier

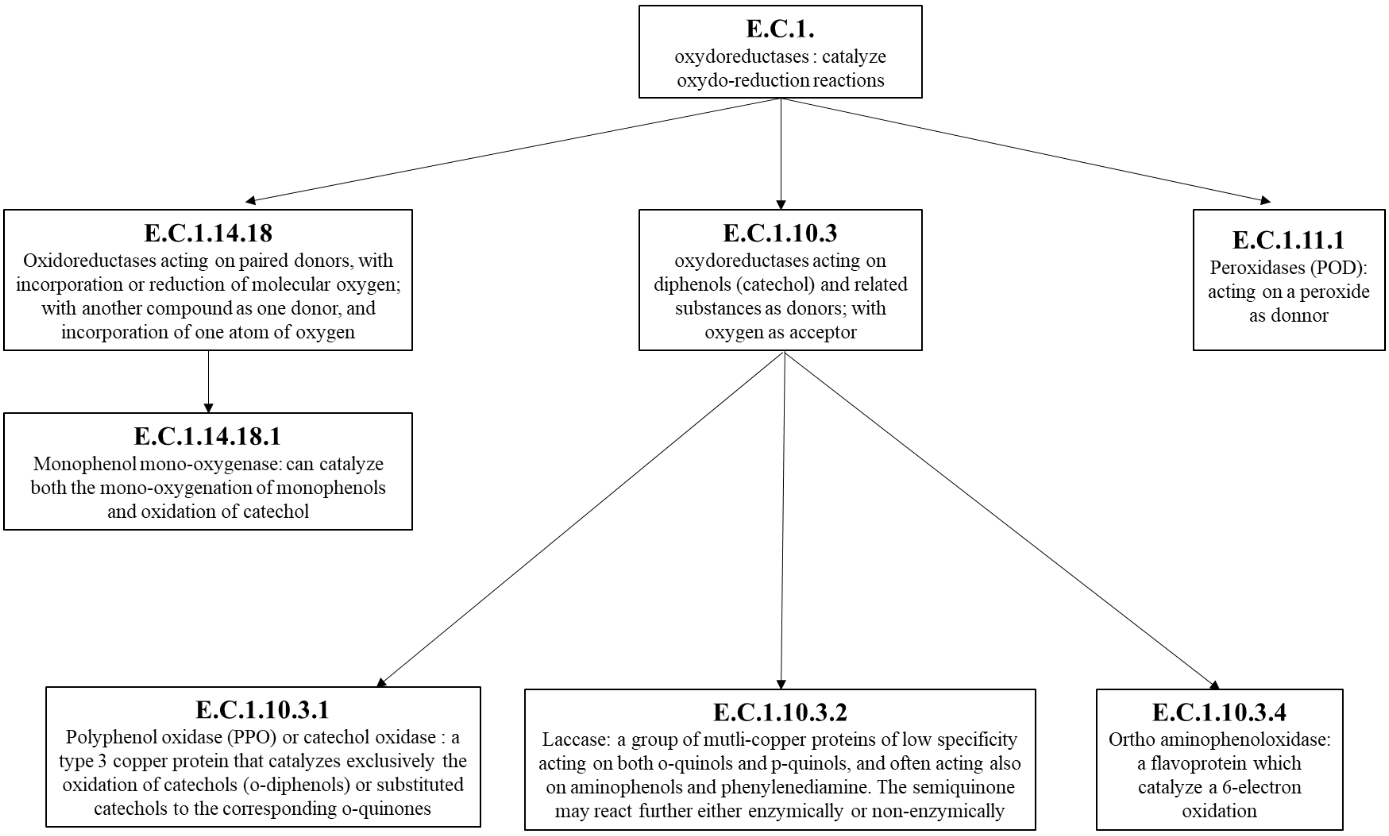

Figure S1*. Classification of enzymes responsible for enzymatic browning*

Table S1. *^{1}H and ^{13}C NMR assignments of compounds N2, N3, N4, N6 and N8. The analyses were carried out in the solvent acetone-d_6*

Compound	ring	H/C position	Chemical shift/ppm (δH)	Multiplicity pattern	J-coupling/Hz	Chemical shift/ppm (δC)
**N2**	**A-u**	4a	-	-	-	100.08
		5 (OH)	8.17	s	-	156.76
		6	5.96	d	2.29 (^{4}J)	95.53
		7 (OH)	8.02	s	-	157.24

		8	5.79	d	2.29 (^{4}J)	95.13
		8a	-	-	-	157.03
	**C-u**	2	4.54	d	2.17 (^{3}J)	67.92
		3	4.03	m	-	67.95
		3 (OH)	4.33	s		
		4*α*	2.89	dd	15.08 (^{2}J) / 5.41 (^{3}J)	28.93
		4β	2.38	dd	15.85 (^{2}J) / 8.79 (^{3}J)	
	**B-u**	1′	-	-	-	130.94
		2′	-	-	-	122.17
		3′ (OH)	7.04	s	-	143.53
		4′ (OH)	7.79	s	-	144.96
		5′	6.76	d	8.34 (^{3}J)	115.00
		6′	6.81	d	8.36 (^{3}J)	118.63
	**A-l**	4a	-	-	-	100.93
		5 (OH)	8.35	s	-	156.49
		6	6.13	s	-	96.71
		7 (OH)	7.47	s	-	155.05
		8	-	-	-	101.96
		8a	-	-	-	153.78
	**C-l**	2	4.52	d	1.89 (^{3}J)	67.96
		3	3.92	m	-	67.91
		3 (OH)	4.08	s	-	
		4*α*	2.93	dd	15.08 (^{2}J) / 5.22 (^{3}J)	28.37
		4β	2.54	dd	16.53 (^{2}J) / 8.77 (^{3}J)	
	**B-l**	1′	-	-	-	131.97
		2′	6.89	d	1.78 (^{4}J)	114.57
		3′ (OH)	7.86	s	-	145.07
		4′ (OH)	7.74	s	-	145.07
		5′	6.70	d	8.09 (^{3}J)	115.09
		6′	6.73	dd	8.79 (^{3}J) / 1.79 (^{4}J)	119.79
**N3**	**A-u**	4a	-	-	-	100.67
		5 (OH)	8.32	s	-	157.18
		6	6.01	d	2.26 (^{4}J)	96.22
		7 (OH)	8.08	s	-	157.79
		8	5.82	d	2.22 (^{4}J)	96.42
		8a	-	-	-	196.92
	**C-u**	2	4.53	d	8.38 (^{3}J)	82.80
		3	3.99	m	-	68.47
		3 (OH)	4.33	s	-	
		4*α*	2.98	dd	16.24 (^{2}J) / 5.89 (^{3}J)	29.42
		4β	2.52	dd	15.81 (^{2}J) / 9.36 (^{3}J)	
	**B-u**	1′	-	-	-	131.57
		2′	7.00	d	1.92 (^{4}J)	116.45

		3′	-	-	-	147.32
		4′ (OH)	7.90	s	-	147.34
		5′	6.805	d	8.14 (^{3}J)	116.45
		6′	6.94	dd	8.06 (^{3}J) / 1.9 (^{4}J)	122.43
	**A-l**	4a	-	-	-	101.69
		5 (OH)	8.32	s	-	153.27
		6	6.15	s	-	96.18
		7 (OH)	8.2	s	-	149.62
		8	-	-	-	124.97
		8a	-	-	-	149.00
	**C-l**	2	4.62	d	7.57 (^{3}J)	82.71
		3	3.96	m	-	68.20
		3 (OH)	4.08	s	-	
		4α	2.90	dd	16.42 (^{2}J) / 5.18 (^{3}J)	28.6
		4β	2.58	dd	16.38 (^{2}J) / 8.23 (^{3}J)	
	**B-l**	1′	-	-	-	131.63
		2′	6.75	d	1.85 (^{4}J)	114.81
		3′ (OH)	7.95	s	-	145.44
		4′ (OH)	7.74	s	-	145.78
		5′	6.67	d	8.12 (^{3}J)	115.58
		6′	6.63	dd	8.15 (^{3}J) / 1.85 (^{4}J)	120.23
**N4-A**	**A-u**	4a	-	-	-	100.71
		5 (OH)	8.30	s	-	157.26
		6	6.02	d	2.33 (^{4}J)	96.16
		7 (OH)	8.08	s	-	157.76
		8	5.9	d	2.29 (^{4}J)	95.5
		8a	-	-	-	154.05
	**C-u**	2	4.57	d	6.13 (^{3}J)	83.15
		3	4.09	m	-	68.83
		3 (OH)	4.34 or under H3C	s	-	
		4α	2.96	dd	16.39 (^{2}J) / 7.12 (^{3}J)	28.91
		4β	2.55	dd	15.53 (^{2}J) / 8.95 (^{3}J)	
	**B-u**	1′	-	-	-	123.79
		2′	6.80	d	2.21 (^{4}J)	114.25
		3′ (OH)	7.65	s	-	146.36
		4′ (OH)	7.30	s	-	143.94
		5′	-	-	-	121.33
		6′	6.85	d	2.24 (^{4}J)	157.08
	**A-l**	4a	-	-	-	100.89
		5 (OH)	8.41	s	-	156.79
		6	6.15	s	-	96.55
		7 (OH)	7.43	s	-	155.05
		8	-	-	-	104.99
		8a	-	-	-	153.84

	**C-l**	2	4.73	d	6.21 (^{3}J)	81.93
		3	4.06	m	-	
		3 (OH)	4.34 or under H3C	s	-	67.66
		4α	2.84	dd	16.63 (^{2}J) / 5.1 (^{3}J)	
		4β	2.62	dd	10.47 (^{2}J) / 6.94 (^{3}J)	28.02
	**B-l**	1′	-	-	-	131.84
		2′	6.84	d	1.83 (^{4}J)	114.82
		3′ (OH)	8.08	s	-	145.45
		4′ (OH)	7.70	s	-	145.59
		5′	6.72	d	8.48 (^{3}J)	115.67
		6′	6.69	dd	8.04 (^{3}J) / 1.92 (^{4}J)	119.31
**N4-B**	**A-u**	4a	-	-	-	100.78
		5 (OH)	8.26	s	-	157.17
		6	6.00	d	2.37 (^{4}J)	96.13
		7 (OH)	8.08	s	-	157.71
		8	5.82	d	2.34 (^{4}J)	95.51
		8a	-	-	-	157.71
	**C-u**	2	4.42	d	8.26 (^{3}J)	83.07
		3	3.82	m	-	
		3 (OH)	4.34 or under H3C	s	-	68.86
		4α	2.96	dd	16.52 (^{2}J) / 8.63(^{3}J)	28.98
		4β	2.46	dd	16.29 (^{2}J) / 9.1 (^{3}J)	
	**B-u**	1′	-	-	-	124.51
		2′	6.82	d	2.08 (^{4}J)	113.30
		3′ (OH)	7.83	s	-	146.65
		4′ (OH)	7.29	s	-	144.45
		5′	-	-	-	122.01
		6′	6.71	d	2.58 (^{4}J)	157.02
	**A-l**	4a	-	-	-	101.03
		5 (OH)	8.42	s	-	156.69
		6	6.15	s	-	96.31
		7 (OH)	7.23	s	-	155.06
		8	-	-	-	104.55
		8a	-	-	-	153.84
	**C-l**	2	4.55	d	5.71 (^{3}J)	82.93
		3	3.99	m	-	
		3 (OH)	4.34 or under H3C	s	-	68.25
		4α	2.93	dd	15.77 (^{2}J) / 6.55(^{3}J)	28.91
		4β	2.59	dd	9.48 (^{2}J) / 6.32 (^{3}J)	
	**B-l**	1′	-	-	-	132.10
		2′	6.94	d	2.00 (^{4}J)	115.08
		3′ (OH)	8.00	s	-	145.38
		4′ (OH)	7.79	s	-	145.61

		5′	6.69	d	8.23 (^3J)	115.60
		6′	6.77	dd	8.16 (^3J) / 2.04 (^4J)	120.05
**N6**	**A-u**	4a	-	-	-	100.66
		5 (OH)	8.23	s	-	157.23
		6	6.03	d	2.04 (^4J)	96.25
		7 (OH)	8.03	s	-	157.80
		8	5.89	d	2.04 (^4J)	95.49
		8a	-	-	-	156.86
	**C-u**	2	4.60	d	8.05 (^3J)	82.63
		3	4.01	m	-	68.36
		3 (OH)	4.14	s	-	
		4α	2.96	dd	9.57 (^2J) / 5.62(^3J)	
		4β	2.55	dd	15.85 (^2J) / 8.64 (^3J)	29.16
	**B-u**	1′	-	-	-	134.69
		2′	6.92	d	1.71 (^4J)	115.72
		3′ (OH)	7.80	s	-	147.25
		4′	-	-	-	147.45
		5′	6.22	d	8.38 (^3J)	115.96
		6′	6.78	dd	8.38 (^3J) / 1.8 (^4J)	119.72
	**A-l**	4a	-	-	-	101.79
		5 (OH)	8.27	s	-	153.33
		6	6.19	s	-	96.17
		7 (OH)	8.17	s	-	149.67
		8	-	-	-	125.03
		8a	-	-	-	149.05
	**C-l**	2	4.66	d	7.32 (^3J)	82.68
		3	3.98	m	-	67.97
		3 (OH)	4.05	s	-	
		4α	2.93	dd	10.17 (^2J) / 5.02 (^3J)	28.60
		4β	2.62	dd	16.34 (^2J) / 7.86 (^3J)	
	**B-l**	1′	-	-	-	131.63
		2′	6.71	d	1.71 (^4J)	114.88
		3′ (OH)	7.83	s	-	146.72
		4′	-	-	-	145.90
		5′	6.73	d	8.11 (^3J)	115.69
		6′	6.64	dd	7.93 (^3J) / 1.59 (^4J)	119.92
**N8**	**A-u**	4a	-	-	-	100.24
		5 (OH)	8.36	s	-	157.41
		6	6.01	d	2.1 (^4J)	95.90
		7 (OH)	7.88	s	-	157.92
		8	5.61	d	2.34 (^4J)	96.98
		8a	-	-	-	155.97
	**C-u**	2	4.06	d	9.58 (^3J)	79.04
		3	4.01	m	-	66.22
		4α	2.95	dd	10.12 (^2J) / 5.40 (^3J)	27.95

	4β	2.53	dd	14.29 (^2J) / 11.08 (^3J)	
**B-u**	1′	-	-	-	89.61
	2′α	2.71	d	12.03 (^2J)	45.20
	2′β	2.49	d	12.03 (^2J)	
	3′ (OH)	5.78	s	-	94.80
	4′	-	-	-	192.69
	5′	6.38	s	-	112.91
	6′	-	-	-	162.57
**A-l**	4a	-	-	-	103.96
	5 (OH)	9.72	s	-	164.46
	6	6.22	s	-	90.84
	7	-	-	-	167.13
	8	-	-	-	105.43
	8a	-	-	-	154.73
**C-l**	2	4.86	d	7.84 (^3J)	83.46
	3	4.12	m	-	67.70
	3 (OH)	4.23	s	-	
	4α	2.98	dd	11.16 (^2J) / 5.40 (^3J)	28.55
	4β	2.95	dd	16.18 (^2J) / 8.41 (^3J)	
**B-l**	1′	-	-	-	131.16
	2′	6.94	d	1.51 (^4J)	115.04
	3′ (OH)	7.97	s	-	145.88
	4′ (OH)	7.92	s	-	146.06
	5′	6.83	d	7.97 (^3J)	116.02
	6′	6.80	dd	7.97 (^3J) / 1.69 (^4J)	119.98

Figure S2 - *2D ¹H/¹³C HMBC, top 1D ¹H, side 1D ¹³C spectrum (a) N2; (b) N3; (c) N4; (d) N6 and (e) N8*

(a)- N2

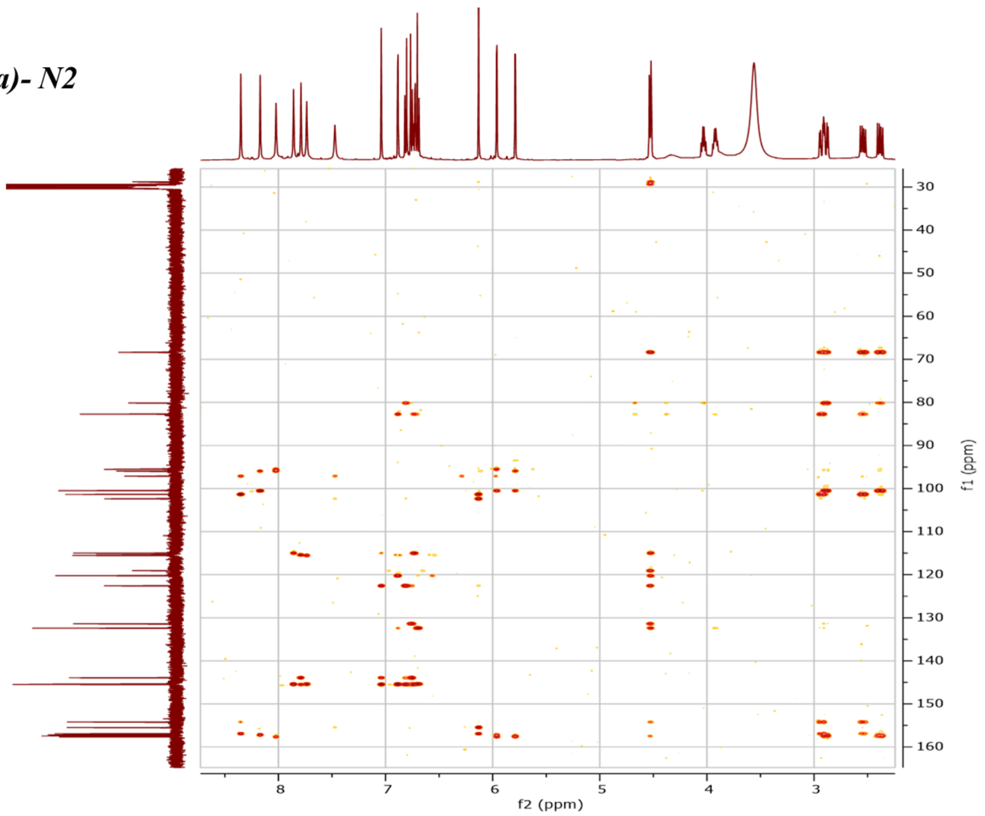

(b) – N3

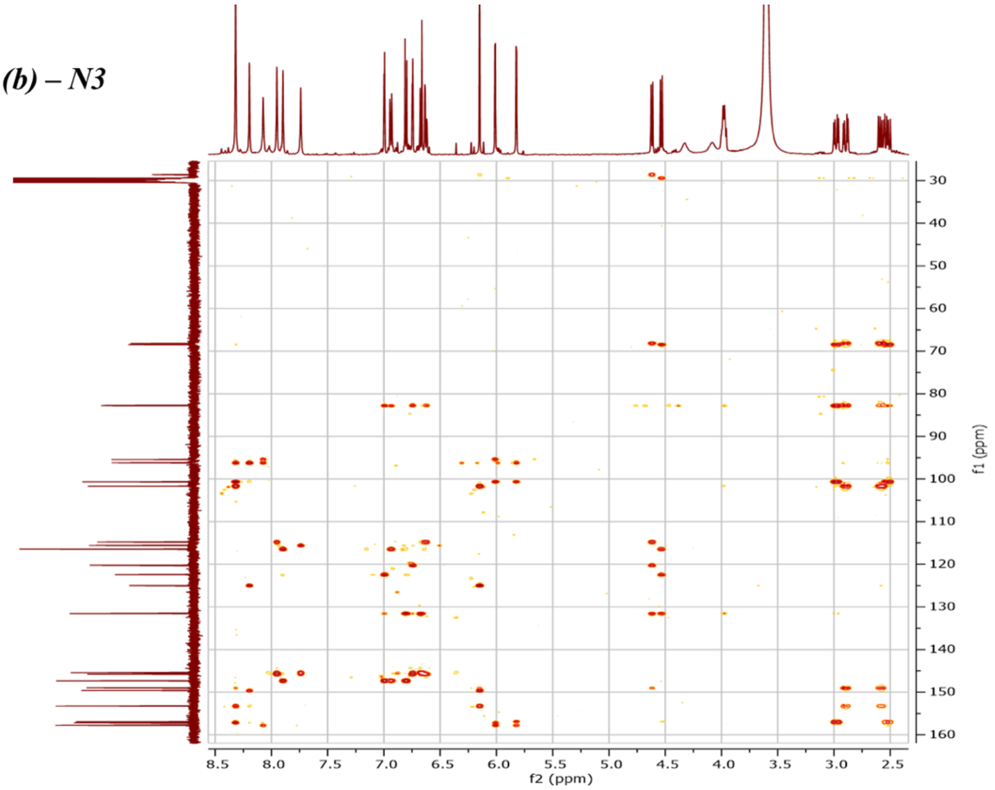

(c) – N4

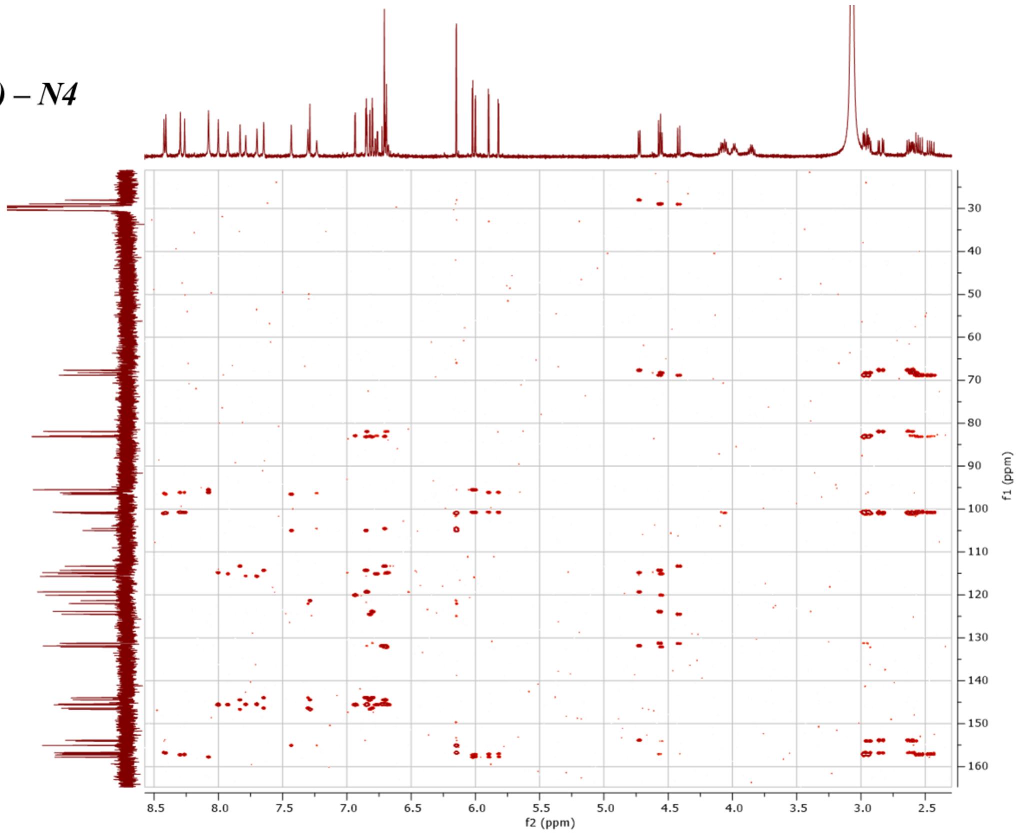

(d) – N6

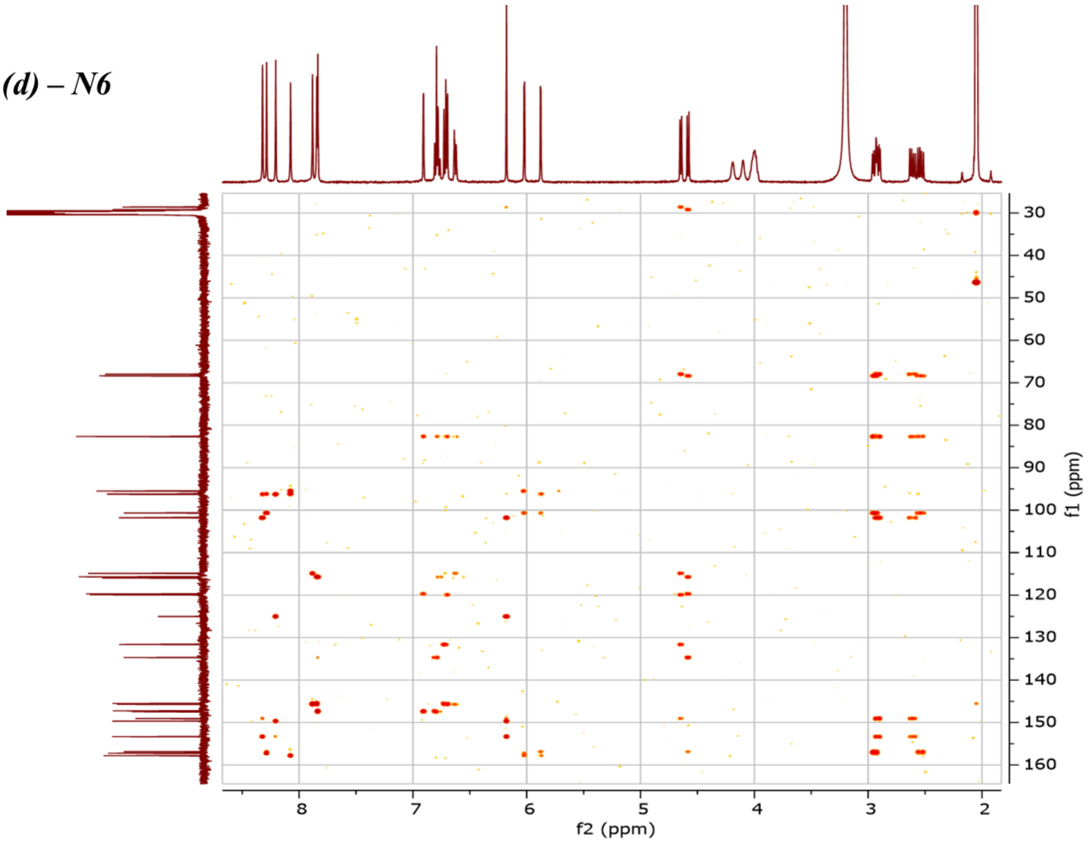

(e) - N8

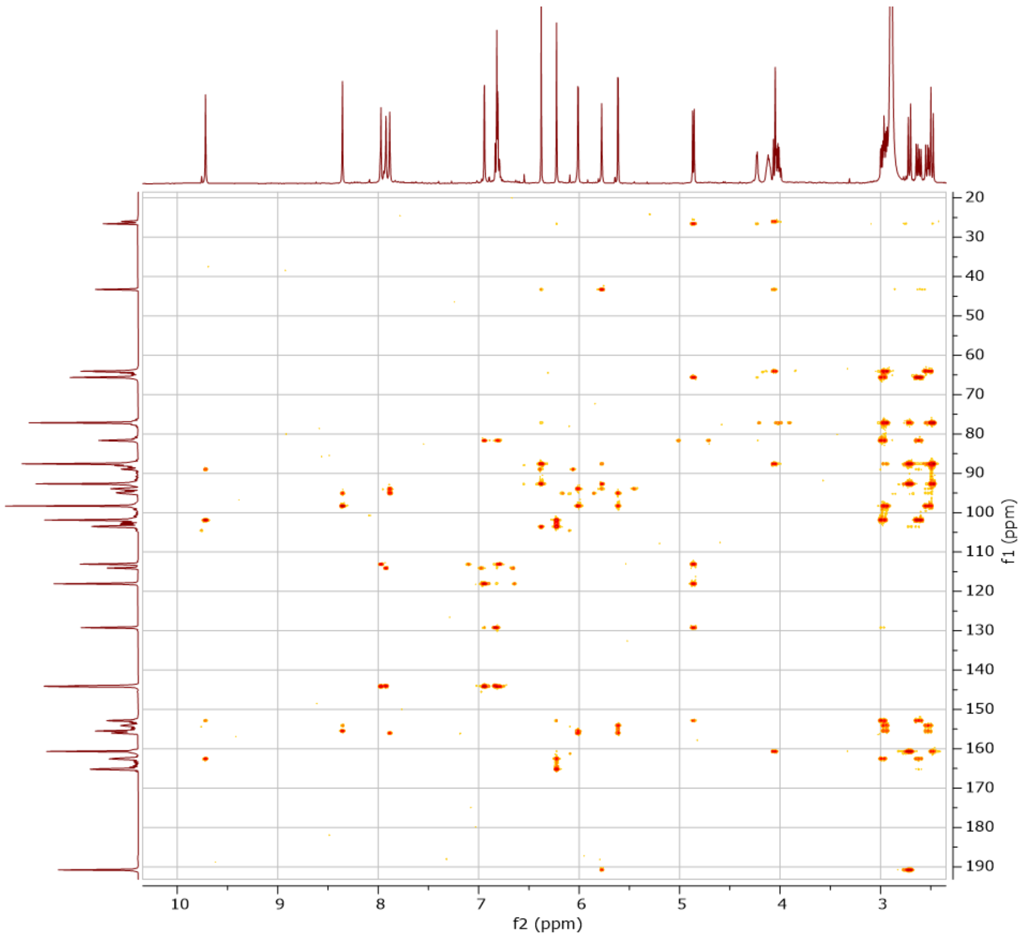

20
30
40
50
60
70
80
90
100
110
120
130
140
150
160
170
180
190
f1 (ppm)
10
9
8
7
6
5
4
3
f2 (ppm)

Figure S3 - *^{1}H 2D ROESY spectrum showing correlations (in blue) between phenolic and aromatic protons, ROE correlations between phenolic protons due to chemical exchange appear in red (a) N2; (b) N3; (c) N4; (d) N6 and (e) N8*

(a)- N2

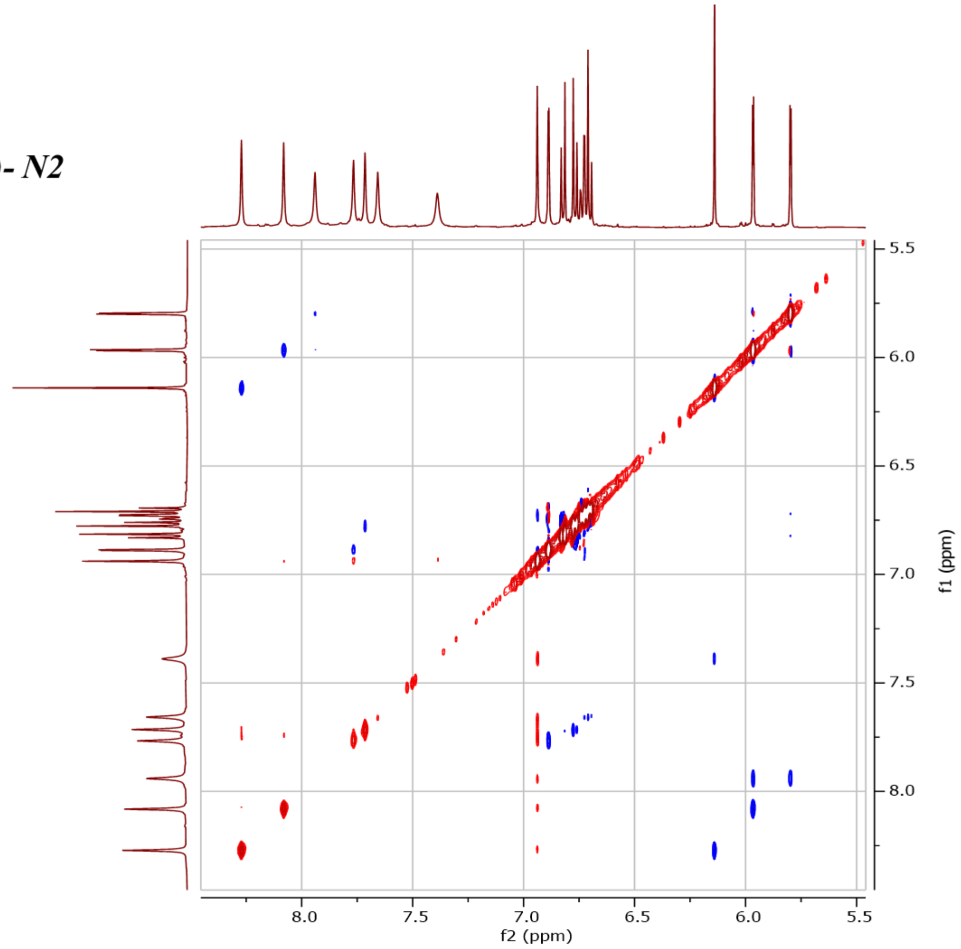

(b) – N3

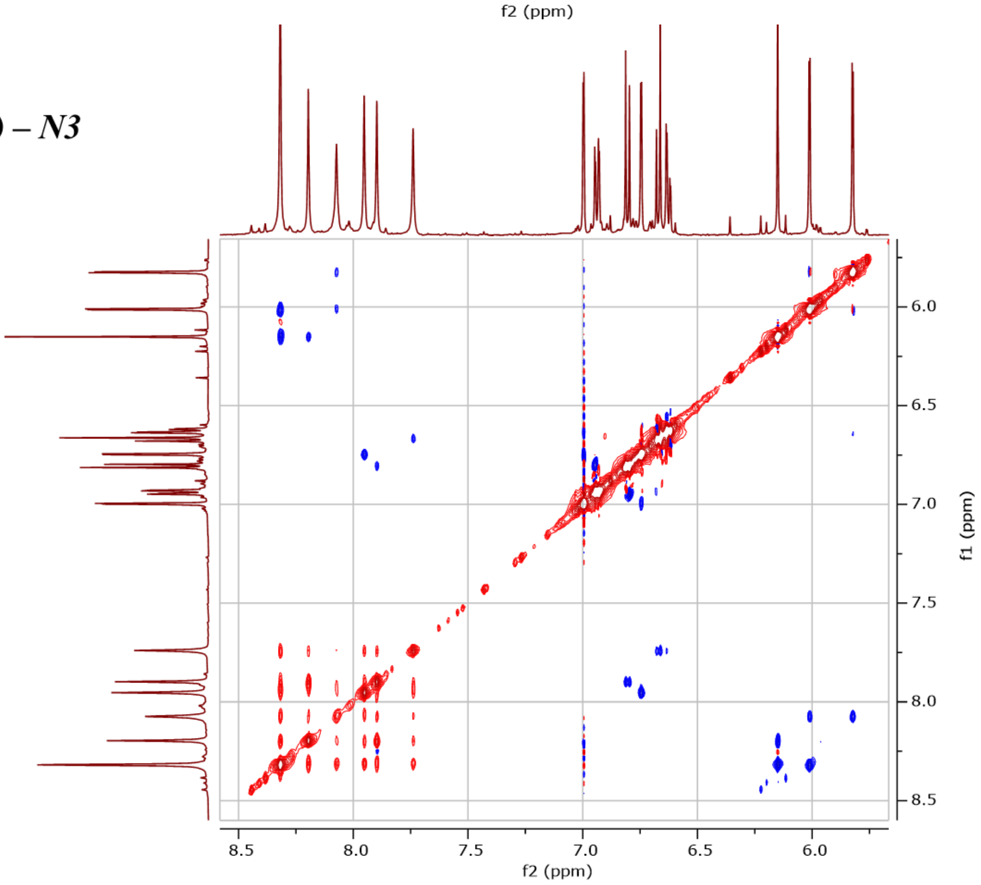

(c) – N4

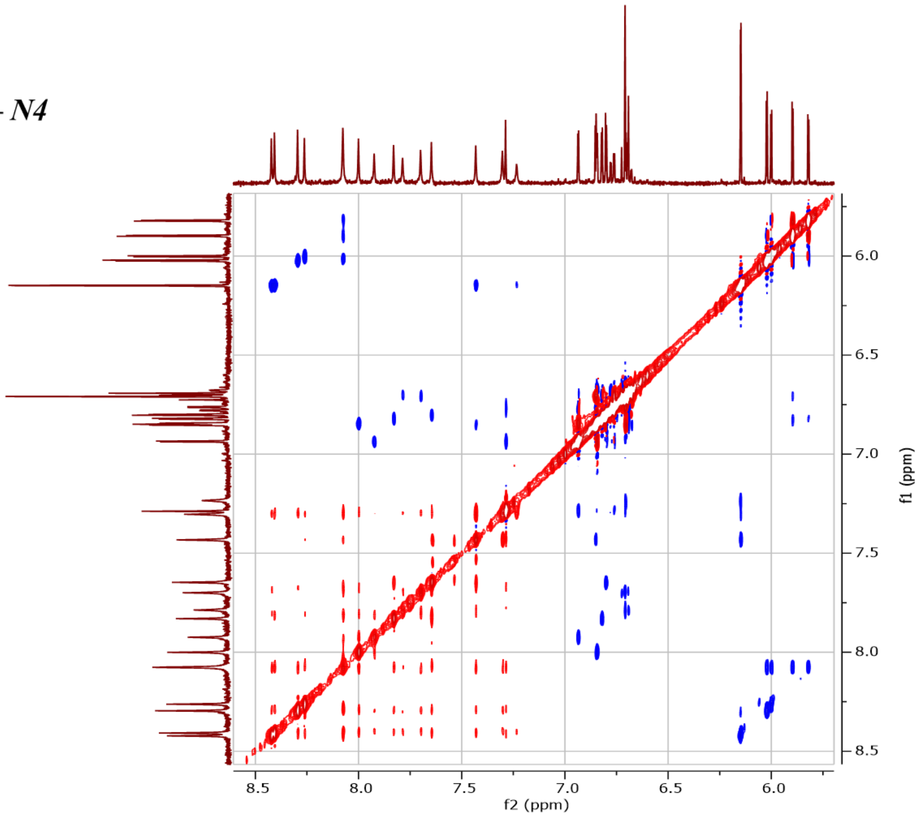

(d) – N6

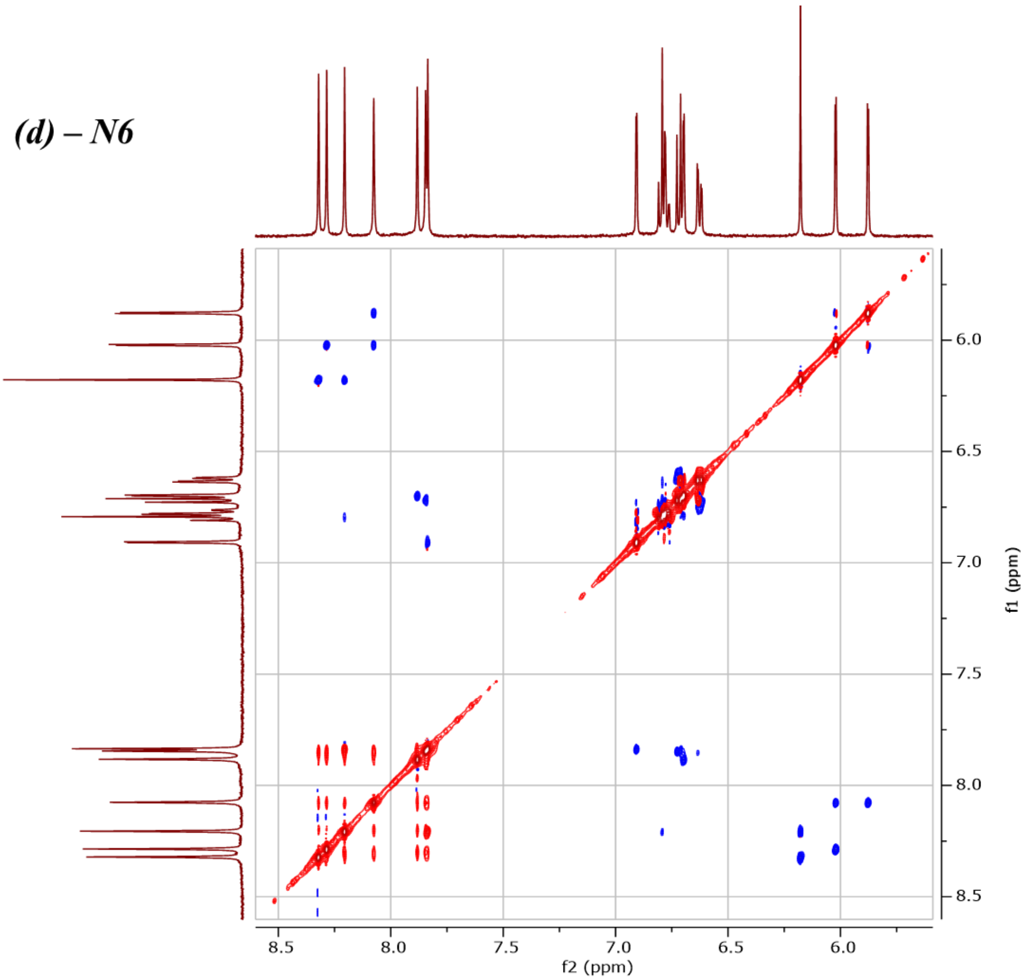

(e) - N8

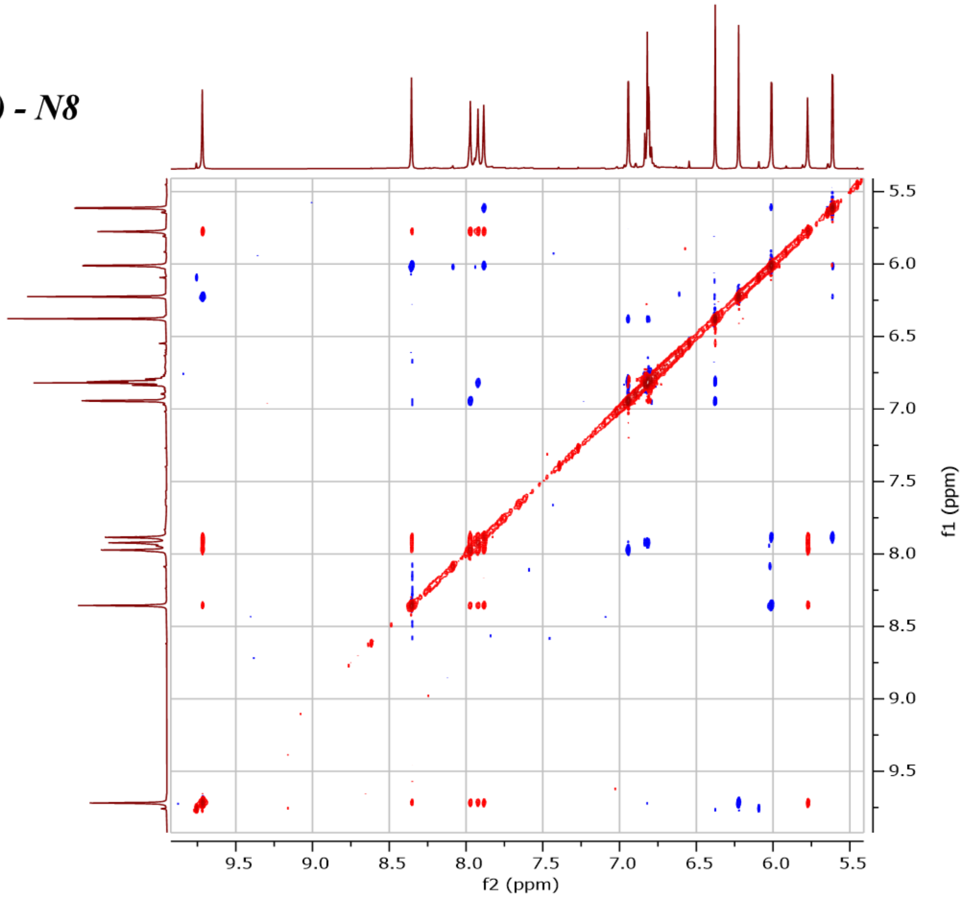

Supplement: Supplementary file 1 [file molecules-26-06165-s001.zip › molecules-1402951-supplementary.pdf]
